# Effect of socioeconomic status on smoking cessation behavior in selected African countries: Secondary analysis of Global Adult Tobacco Survey data (2014–2018)

**DOI:** 10.1371/journal.pone.0274746

**Published:** 2022-09-19

**Authors:** Zinto Gabsile Vilane, Prakash Babu Kodali, Kavumpurathu Raman Thankappan

**Affiliations:** Department of Public Health and Community Medicine, Central University of Kerala, Kasaragod, Kerala, India; Chung Shan Medical University, TAIWAN

## Abstract

**Introduction:**

Tobacco use remains a global public health challenge. While studies report that smoking cessation reduces the risk of cancer and other NCDs, evidence is scarce in African region on socio-economic determinants of smoking cessation behavior. This study examined the socio-economic differentials of smoking cessation behavior among smokers in four African countries.

**Methods:**

The study was conducted through secondary analysis of Global Adult Tobacco Survey (GATS) data from four African countries (Ethiopia, Kenya, Senegal and Tanzania). Smoking cessation behavior was assessed using two variables i) intention to quit smoking in next 12 months and ii) previous quit attempts made within 12 months preceding the survey. The weighted percentages for intention to quit smoking and previous quit attempts were computed. The adjusted odds ratios were computed using multinomial logistic regression to identify the association between socio-economic factors and smoking cessation behavior.

**Results:**

Across the four countries studied, the previous quit attempts among smokers were in the range of 39.6% to 53.7%. Around 7.6% to 15.8% of the smokers tried to quit with an assistance. In Ethiopia over 76.5% of current smokers reported no intention to quit in next 12 months after survey, whereas the same was 50.4% in Senegal. While country specific differences were observed, females, those belonging to the poorest wealth index, unemployed and those without any formal education reported significantly lower odds of previous quit attempts or having an intention to quit smoking.

**Conclusion:**

The socio-economic vulnerabilities were found to compromise smoking cessation behavior among the smokers in countries studied. Targeted interventions, adherence to smokefree laws, and provision of cessation support are essential to improve quit rates and mitigate tobacco risks among socio-economically vulnerable population.

## Introduction

Tobacco use remains a major public health problem globally as it is associated with increased risk of developing chronic diseases. The Global Burden of Disease (GBD) Collaborators (2021) reported that there were 1.14 billion smokers in 2019 [1]. In 2019, tobacco use resulted in 7.69 million deaths and 200 million disability-adjusted life-years. Approximately 80% of tobacco users are from low-income and middle-income countries [2]. It is estimated that by 2030 there will be 10 million deaths attributed to tobacco use, with over 70% of the deaths from developing countries. In the African region the death rate attributed to tobacco increased by 70% from 1990 to 2016 [3].

Though the prevalence of smoking showed a decline globally, there are still exceptions with the Eastern Mediterranean and African region [4]. The fifteen-year (2000–2015) data from World Health Organization (WHO) indicates an increase in smoking prevalence in 27 countries of which 15 are in Sub-Saharan Africa. According to the second round of Global Adult Tobacco Survey (GATS), the prevalence of smoking among individuals aged 15 years and above was 11.6% in Kenya, 6.0% in Senegal, 5.0% in Ethiopia and 8.7% in Tanzania [5].

Quitting smoking lowers the risk of major Non-Communicable Diseases (NCD) like cardiovascular diseases, diabetes, chronic lung diseases and cancer. The American Cancer Society (2020), estimates that quitting smoking before the age of 40 years effectively helps to reduce the risk of dying from smoking related diseases by 90%. The smoking cessation is defined as a process by which a smoker discontinues using tobacco with or without support, eventually quitting its use [6]. Smoking cessation is known to be largely influenced by the individual’s voluntary intention to quit smoking, and attempts made towards quitting smoking [7]. Evidence suggests that the smoking cessation behavior (reflected by a smoker’s previous quit attempts and intention to quit) is an indicator for future quit attempts and their success [8]. Moreover, literature suggests that smokers when assisted to quit smoking have a greater odds of undergoing successful quit attempts [8–10]. Assistance is the key for successful quitting since attempts to quit smoking without adequate support have a minimal success rate, as only 3–5% self-quitters succeed in long-term [11]. The global coverage of population by at least one tobacco control measure has increased drastically since 2007. As of 2019 at least 65% of the population is covered comprehensively for tobacco control [12]. However, studies argue that even though smoking prevalence is declining in the general population, it is gradually becoming concentrated in groups that are either socially and/or economically underprivileged [13].

From a social determinants perspective, the individual’s vulnerability to health compromising conditions could be attributed to several social & structural determinants such as policies, income, education, societal and cultural values [14]. Existing literature suggests that socio-economic determinants significantly influence smoking behavior. Studies reported that smoking behavior varied with countries across the determinants such as gender, place of residence, age, education, occupation and wealth index [15]. An earlier study from Lebanon reported that female smokers had low intention to quit smoking [16]. A recent study on female smokers in South Korea reported that intention to quit was strongly predicted by smokers’ awareness of smoking cessation policy and cessation support [17]. Another study from the United States reported that smokers with less than high school education were less likely to make an attempt to quit smoking [18], indicating that belonging to socially vulnerable groups such as being female, unable to read or write negatively impacts smoking cessation behavior. Moreover, studies observe socio-economic disadvantage (such as belonging to racial/ethnic minorities, poor, and unemployed) reduces the access to smoking cessation aides [19]. Internationally it is recognized that tobacco quit rates adhere to a steep socio-economic gradient. Developing countries have poorer tobacco quit rates than developed nations. Moreover, stark within country differentials in quit behavior are observed in developing nations [20].

Limited evidence exists on socio-economic determinants of smoking cessation behavior in the African region. A recent systematic review on tobacco cessation interventions in LMICs report a shortage of evidence on smoking cessation in context of African countries [21], unavailability of representative data on smoking cessation is regarded as one of the challenges towards effective smoking cessation strategies in Africa [22]. While few surveys reported a lack of awareness and access to smoking cessation support among vulnerable populations [23, 24], surveys of smoking cessation usually cover urban, selected regions or certain population subgroups and are rarely nationally representative. Therefore, we conducted this study with the following objectives.

1. To examine the smoking cessation behavior (previous quit attempts and intention to quit) among adult smokers in four African Countries (Ethiopia, Kenya, Senegal, & Tanzania)
2. To examine the relationship between socio-economic status (SES) and smoking cessation behavior in these four African Countries
3. To assess the between differences among these four countries in associations between SES and smoking cessation behavior.

## Materials and methods

### Data source

We analyzed the recent Global Adult Tobacco Survey (GATS) data from four countries namely Kenya (2014), Senegal (2015), Ethiopia (2016) and Tanzania (2018). The population of the countries during the year when GATS was conducted was 46.7 million in Kenya, 14.58 million in Senegal, 103.6 million in Ethiopia and 56.32 million in Tanzania. GATS is a nationwide representative survey that applies uniform and customary instructions and methods among all countries globally undertaking the survey. The GATS data in SPSS format were obtained from the Centers for Disease Control and Prevention (CDC) website (https://nccd.cdc.gov/GTSSDataSurveyResources/Ancillary/DataReports.aspx?CAID=2). GATS survey interviewed 4408 individuals from Kenya, 4347 individuals from Senegal, 10150 individuals from Ethiopia and 4797 individuals from Tanzania.

### Study sample

The data from Global Adult Tobacco Survey (GATS) round 2 for individual countries were accessed and cleaned. The sample weights were applied. In connection to study objectives the data of smokers were extracted. The final sample comprised of 702, 449, 272 and 378 current smokers from Ethiopia, Kenya, Senegal and Tanzania respectively.

### Data collection tools and techniques

The GATS surveys are conducted using standard tools and procedures to maintain uniformity and comparability across the countries. The global standardized survey questionnaire used by GATS captures information on participants’ demographic data, tobacco usage, passive smoking, cessation behavior and associated factors, economics, media, knowledge and attitudes towards the use of tobacco. The survey questionnaire was designed to be administered using structured interview technique. The details of tools and other resources used by GATS are available at following official weblink of CDC https://nccd.cdc.gov/GTSSDataSurveyResources/Ancillary/Documentation.aspx?SUID=1&DOCT=1.

#### Study variables

*Previous quit attempts*. In context of this study, the “previous quit attempts” were defined as any attempts made by the current smoker to quit tobacco in past 12 months preceding the survey. The previous quit attempts were categorized under three responses “No attempt”, “Tried quitting without assistance” and “Tried quitting with assistance”. In the GATS survey questionnaire, the variables “D01” asked as “*During the past 12 months have you tried to stop smoking*?” and eight variables under “D03” ranging from D03A to D03G1 (asked as *During the past 12 months*, *did you use any of the following to try to stop smoking tobacco*? ‥ ‥ ‥) were used to compute the variable.

*Intention to quit within next 12 months*. In the study’s context “intention to quit” was defined as the self-reported intention of the current smoker to quit tobacco within the next 12 months following the survey. In the GATS survey questionnaire, the variable “D08” read as “*Which of the following best describes your thinking about quitting smoking*? *I am planning to quit within the next month*, *I am thinking about quitting within the next 12 months*, *I will quit someday but not within the next 12 months*, *or I am not interested in quitting*?” was used. The responses were recoded into three categories to fit to the study variable. The recoded responses were “No intention to quit”, “Intention to quit within one month” and “Intention to quit within 12 months”

Additionally, under socio-economic status the variables such as gender (male/female), age groups (15–35 years, 36–50 years, 51 years and more), education level (no education, primary, secondary education and above), place of residence (rural/urban), marital status (married/unmarried), and wealth index (poorest, poor, middle, rich, richest) were included as variables in the study. The variables such as education level, marital status and occupation status were recoded from existing variables, the variable wealth index was computed as a composite index of 13 variables (i.e., variables A06A to A06N in the GATS questionnaire) capturing the amenities and assets owned by the household.

### Data cleaning & analysis

In data cleaning process the data were cleaned and prepared for analysis as per study objectives. New variables (such as wealth index, previous quit attempts etc.,) were computed and existing variables were recoded. Sample weights were applied as per standardized norms.

Data analysis was performed using the International Business Machine (IBM) Statistical Package of Social Sciences (SPSS) (version 27.0 for Windows, IBM Corporation, Chicago, Illinois, United States). Descriptive statistics were generated for all variables and comparisons between countries were made. Multinomial logistic regression was used to identify the predictive association of socio-economic factors with previous quit attempts and intention to quit smoking. It was ensured that the assumptions of multinomial logistic regression such as i) nominal dependent variable, ii) independence among dependent variable choices, and iii) no multi-collinearity were adhered. The multi-collinearity was assessed using Variance Inflation Factor (VIF). The VIF of independent variables raged between 1.00 to 2.30 across the countries studied, indicating the low multi-collinearity. A p-value of less than or equal to 0.05 was considered as statistically significant. Adjusted odds ratios (AOR) and 95% confidence intervals were computed reflecting the predictive association.

Comparisons across the countries with respect to intention to quit and previous quit attempts among the smokers were made. The socio-economic predictors associated with intention to quit and previous quit attempts among smokers were identified and compared. To address for potential confounding bias, adjusted odds ratios were computed using multinomial logistic regression. The variables with significant adjusted odds ratios obtained from multinomial logistic regression were assessed for possibility of effect modification by checking for two-way interaction between potential effect modifiers. The presence of effect modification was found to be minimal, and the odds calculated addressing two-way interaction between independent variables concurred with odds for the country specific model. The detailed tables outlining the adjusted odds computed after addressing for the two-way interaction are reported in S1 File.

### Ethical consideration

The original GATS survey followed the due procedures of ethical approval, informed consent, confidentiality and voluntary participation. Informed consent in writing was obtained from all the participants aged 18 years and above. For minor participants (aged 15–17 years), parental consent and assent from the minor were obtained prior to survey. Confidentiality was ensured by deidentifying the data. The standard protocol for data collection during GATS surveys is given at https://nccd.cdc.gov/GTSSDataSurveyResources/Ancillary/Documentation.aspx?SUID=1&DOCT=1. For the purpose of this study, the informed consent was waived as the study was conducted as a secondary analysis of deidentified micro data obtained from the GATS survey official website. The conduct of the study was approved by Institutional Human Ethics Committee of Central University of Kerala (IHEC/CUK/2021/08).

## Results

Kenya had the highest proportion of current smokers (7.8%) followed by Tanzania (6.8%), Senegal (5.4%) and Ethiopia (3.7%). Across the four countries studied, more than 83% of current smokers were males. Moreover, a significant percent of the current smokers belonged to poorest or poor wealth quintile. Table 1 outlines the characteristics of the smokers across the countries.

**Table 1:** Percentage of smokers across various socio-economic characteristics in the GATS sample.

	Ethiopia (N = 10150)	Kenya (N = 4408)	Senegal (N = 4347)	Tanzania (N = 4797)
**Gender**	% (95% CI)	% (95% CI)	% (95% CI)	% (95% CI)
Female	0.6(0.5–0.7)	0.4(0.3–0.5)	0.2(0.1–0.3)	0.6(0.5–0.7)
Male	3.1(2.9–3.2)	7.4(7.0–7.7)	5.2(4.7–5.7)	6.2(5.9–6.5)
**Residence**				
Rural	0.8(0.7–0.9)	5.3(5.0–5.6)	2.5(2.2–2.9)	4.7(4.5–4.9)
Urban	2.9(2.7–3.0)	2.5 (2.5–2.3)	2.8(2.5–3.2)	2.1(1.9–2.3)
**Age Group**				
15 years-35 years	1.8(1.7–1.9)	3.0(2.8–3.3)	2.6(2.2–2.9)	2.4(2.2–2.5)
36–50 years	1.0(0.9–1.1)	2.8(2.6–3.1)	1.7(1.4–2.0)	2.7(2.5–2.8)
51 years and more	0.8(0.7–0.9)	1.9(1.7–2.1)	1.2(0.9–1.4)	1.7(1.5–1.8)
**Marital Status**				
Married	2.9(2.8–3.0)	5.4 (5.1–5.7)	3.6(3.2–4.0)	0.6(0.5–0.7)
Unmarried	0.8(0.7–0.8)	2.4(2.2–2.6)	1.8(1.5–2.1)	6.2(5.9–6.5)
**Wealth Index**				
Poorest	1.2(1.1–1.3)	0.7(0.6–0.8)	0.6(0.5–0.8)	1.1(1.0–1.2)
Poor	0.8(0.7–0.9)	1.5(1.3–1.6)	1.4(1.2–1.7)	1.3(1.2–1.5)
Middle	0.9(0.8–1.0)	1.7(1.5–1.9)	0.7(0.5–0.8)	1.4(1.3–1.6)
Rich	0.4(0.3–0.4)	2.1(1.9–2.3)	1.4(1.2–1.7)	1.2(1.0–1.3)
Richest	0.4(0.3–0.4)	1.8(1.6–2.0)	1.2(1.0–1.5)	1.7(1.6–1.9)
**Education**				
No formal Education	1.5(1.4–1.6)	3.6(3.3–3.8)	2.7(2.3–3.0)	2.9(2.7–3.1)
Primary Education	1.4(1.3–1.5)	2.5(1.5–2.7)	1.5(1.3–1.8)	3.1(2.9–3.3)
Secondary Education and Above	0.8(0.7–0.9)	1.7(1.5–1.9)	1.2(1.0–1.4)	0.8(0.7–0.9)
**Occupation**				
Employed	3.2(3.0–3.3)	5.4(5.1–5.7)	4.3(3.8–4.7)	5.9(5.7–6.2)
Unemployed	0.5(0.5–0.6)	2.4(2.2–2.6)	1.1(0.9–1.4)	0.9(0.8–1.0)
**Weighted percentage of smokers**	3.7%(3.5–3.8)	7.8% (7.4–8.1)	5.4%(4.9–5.9)	6.8%(6.7–6.8)

*The percentages reported are weighted percentages.

The cross-country differentials in Intention to Quit and Previous Quit Attempts among the smokers were observable. More than 50% of the smokers from Senegal and Kenya had made quit attempts in the last 12 months preceding survey. However, the same was between 39.6 to 43.3% in Tanzania and Ethiopia. Interestingly, in Tanzania and Kenya a greater percentage of smokers (> 15%) attempted to Quit smoking with an assistance, whereas the least was in Senegal (7.6%). Similar differentials were observed with respect to intention to quit smoking within 12 months. While close to 45% of the smokers in Senegal and Kenya reported intention to quit smoking in next 12 months, the same was around 36% in Tanzania and 23.5% in Ethiopia. The detailed outline of the differentials in previous quit attempts and intention to quit smoking are provided in Table 2.

**Table 2:** Smoking cessation behavior among current smokers across four African countries.

	Ethiopia (N = 702)	Kenya (N = 449)	Senegal (N = 272)	Tanzania (N = 378)
**Previous Quit Attempts**	% (95% CI)	% (95% CI)	% (95% CI)	% (95% CI)
Did not try to Quit	60.2(58.3–62.1)	49.5(47.1–51.9)	46.5(41.8–51.3)	56.9(54.8–59.1)
Tried to Quit without assistance	29.7(27.9–31.5)	34.7(32.4–37.0)	46.1(41.3–50.8)	27.9(25.9–29.9)
Tried to Quit with assistance	10.1(9.0–11.3)	15.8(14.1–17.6)	7.4(5.2–10.2)	15.2(13.7–16.8)
**Intention to Quit with next 12 months**				
No intention to Quit	76.5(74.8–78.2)	56.8(54.4–59.2)	50.1(45.3–54.9)	63.3 (61.2–65.5)
Intend to Quit in One month	12.8(11.6–14.2)	26.1(24.0–28.2)	29.1(24.9–33.6)	16.6(15.0–18.3)
Intend to Quit within 12 months	10.7(9.5–11.9)	17.1(15.4–19.0)	20.8(17.1–24.8)	20.1(18.3–21.8)

*The percentages reported are weighted percentages.

Two multinomial logistic regression models were developed with “Intention to Quit Smoking in the next 12 months” and “Previous Quit Attempts in the last 12 months” as the respective dependent variables. The assumptions of the multivariate analysis were adhered prior to running the model. Adjusted Odds Ratios (AOR) with 95% Confidence Intervals (CI) were computed. A p-value of ≤ 0.05 was considered statistically significant.

The intention to quit varied with different variables across the countries. The wealth index was a significant predictor for intention to quit across all the four countries studied. In Ethiopia, the smokers in the richest quintile had 10 times more odds (AOR = 10.59, 95% CI = 6.11–18.33) of intention to quit within a month compared to smokers from poorest quintile. In Kenya, the smokers of richest quintile had 2.40 times greater odds of intention to quit within next 12 months (AOR = 2.40, 95% CI = 1.37–4.20) compared to their poorest counterparts. In Senegal, smokers in the middle wealth index quintile had 4.38 times greater odds of intention to quit within a month, whereas in Tanzania, smokers in the rich quintile had 1.49 times greater odds of intention to quit within next 12 months compared to smokers from poorest wealth quintile. Similar significant associations were observed across the other predictors such as gender, age group, place of residence, education level and occupation. A detailed outline of the influence of various socio-economic determinants on intention to quit across the countries are given in Table 3.

**Table 3:** Socio-economic factors associated with intention to quit across the four African countries.

	Tanzania		Senegal		Kenya		Ethiopia	
	Intention to Quit		Intention to Quit		Intention to Quit		Intention to Quit	
	Intend to Quit within a month	Intend to quit within 12 months	Intend to Quit within a month	Intend to quit within 12 months	Intend to Quit within a month	Intend to quit within 12 months	Intend to Quit within a month	Intend to quit within 12 months
**Gender**								
Female	0.11(0.05–0.25)**	0.60(0.38–0.95)*	0.86(0.25–2.93)	0.23(0.02–2.69)	1.14(0.63–2.01)	2.01(1.11–3.63)*	5.56(3.00–10.30)**	0.51(0.32–0.82)*
Male (ref)								
**Age Group**								
15 years-35 years	0.90(0.64–1.28)	1.76(1.28–2.42)*	1.64(0.82–3.29)	0.83(0.38–1.81)	1.50(1.04–2.14)*	1.09(0.72–1.67)	0.46(0.29–0.72)*	16.30(6.70–39.64)*
36–50 years	1.17(0.85–1.61)	1.22(0.89–1.68)	0.81(0.39–1.67)	0.85(0.40–1.81)	1.55(1.10–2.19)*	1.70(1.15–2.53)*	0.53(0.30–0.95)*	7.31(2.95–18.10)*
51 years and more (ref)								
**Residence**								
Urban	0.26(0.18–0.38)*	0.78(0.59–1.04)	0.60(0.31–1.17)	0.88(0.41–1.91)	1.51 (1.14–2.00)*	1.24 (0.89–2.69)	8.26(5.09–13.39)	4.17(2.74–6.35)
Rural (ref)								
**Marital Status**								
Married	1.51(1.00–2.27)	1.34(0.89–2.02)	0.78(0.42–1.42)	1.31(0.66–2.57)	1.76(1.30–2.41)*	1.05(0.74–1.51)	1.80(1.08–2.98)*	1.99(1.33–2.96)*
Unmarried(ref)								
**Wealth Index**								
Richest	1.32(0.81–2.16)	0.84(0.51–1.37)	1.28(0.40–4.07)	1.22(0.38–3.90)	2.46(1.52–3.97)*	2.40(1.37–4.20)**	10.59(6.11–18.33)*	1.24(0.71–2.18)
Rich	0.79(0.54–1.19)	1.49(1.03–2.17)*	1.89(0.67–5.29)	1.28(0.43–3.79)	0.83(0.49–1.39)	1.48(0.80–2.73)	14.83(7.78–28.28)*	3.12(1.94–4.99)
Middle	1.15(0.76–1.72)	1.59(1.06–2.37)*	4.38(1.43–13.43)*	1.72(0.49–5.94)	0.89(0.63–1.28)	1.94(1.25–3.00)*	3.12(1.79–5.42)*	0.94(0.60–1.48)
Poor	0.80(0.53–1.20)	1.23(0.83–1.82)	3.55(1.39–9.08)*	0.92(0.32–2.69)	2.09(1.50–2.93)*	4.03(2.67–6.09)*	2.50(1.34–4.68)*	0.81(0.51–1.29)
Poorest (ref)								
**Education**								
No formal Education	1.60(0.92–2.80)	1.33(0.85–2.08)	0.85(0.38–1.92)	0.45(0.20–0.99)*	2.60(1.67–4.05)**	1.77(1.13–2.77)*	0.19(0.07–0.52)**	2.15(1.26–3.66)*
Primary Education	1.62(0.95–2.77)	1.29(0.85–1.97)	1.22(0.56–2.65)	0.58(0.27–1.24)	3.55(2.32–5.43)**	1.14(0.72–1.78)	7.08(4.21–11.92)*	0.94(0.58–1.56)
Secondary Education and Above (ref)								
**Occupation**								
Employed	0.62(0.43–0.90)*	1.04(0.71–1.53)	1.43(0.70–2.96)	1.07(0.74–3.78)	0.60(0.45–0.79)*	0.53(0.38–0.74)*	2.78(1.44–5.36)**	0.52(0.34–0.78)**
Unemployed(ref)								

**Dependent Variable:** Intention to quit smoking in the next 12 months:—*No intention to quit (ref);* Intend to quit within one month; Intention to quit in next 12 months. Ref = Reference Category.

* = p value < 0.05,

** = p value < 0.01.

The previous quit attempts in preceding 12 months varied across the independent variables and countries. Across the countries, females had lower odds of attempting to quit in the preceding one year of the survey. For example, the AOR among females for attempting to quit without assistance was 0.48 (95% CI = 0.31–0.75) in Tanzania and 0.49 (95% CI = 0.27–0.90) in Kenya. The smokers residing in urban regions in Tanzania & Senegal had significantly lower odds for attempting to quit with assistance (AOR = 0.61, 95% CI = 0.44–0.84) and AOR = 0.28 (0.09–0.91) respectively) compared to those who were in rural areas. Whereas in Kenya, and Ethiopia the urban residents had significantly higher odds of having previous quit attempts without assistance (AOR = 1.65, 95% CI = 1.28–2.13 and AOR = 2.72, 95% CI = 1.97–3.75 respectively). Similar significant observations were made with respect to age group, marital status, wealth index, education and occupational status. The detailed results outlining the AORs and 95% CI reflecting influence of various socio-economic variables on previous quit attempts across the countries are given in Table 4.

**Table 4:** Socio-economic factors associated with previous quit attempts across the four African countries.

	Tanzania		Senegal		Kenya		Ethiopia	
	Previous Quit Attempts		Previous Quit Attempts		Previous Quit Attempts		Previous Quit Attempts	
	Tried to quit without assistance	Tried to quit with assistance	Tried to quit without assistance	Tried to quit with assistance	Tried to quit without assistance	Tried to quit with assistance	Tried to quit without assistance	Tried to quit with assistance
**Gender**								
Female	0.48(0.31–0.75)**	0.72(0.41–1.24)	0.94(0.30–3.00)	0.96(0.29–2.59)	0.49(0.27–0.90)*	1.47(0.83–2.61)	0.59(0.40–0.87)*	0.014(0.002–0.085)**
Male (ref)								
**Age Group**								
15 years-35 years	1.65(1.24–2.20)*	1.10(0.75–1.62)	0.97(0.52–1.79)	4.43(1.17–16.73)	1.18(0.85–1.63)	0.90(0.59–1.38)	1.34(0.97–1.86)	20.06(8.24–48.84)*
36–50 years	1.65(1.25–2.19)*	1.90(1.34–2.70)	0.93(0.50–1.73)	1.47(0.36–3.98)	1.36(1.01–1.84)*	1.02(0.67–1.55)	1.50(1.03–2.18)*	6.41(2.41–17.06)**
51 years and more (ref)								
**Residence**								
Urban	0.81(0.63–1.05)	0.61(0.44–0.84)**	0.73(0.40–1.35)	0.28(0.09–0.91)*	1.65(1.28–2.13)*	1.28(0.90–1.80)	2.72(1.97–3.75)*	3.55(2.27–5.56)*
Rural (ref)								
**Marital Status**								
Married	0.80(0.54–1.20)	1.03(0.66–1.61)	0.72(0.42–1.23)	3.48(1.06–11.43)*	1.62(1.21–2.17)*	0.50(0.35–0.71)**	1.65(1.16–2.32)*	2.12(1.31–3.42)*
Unmarried(ref)								
**Wealth Index**								
Richest	0.89(0.60–1.32)	0.80(0.46–1.39)	0.73(0.28–1.90)	0.50(0.08–3.03)	2.78(1.79–4.23)**	0.61(0.32–1.15)	2.45(1.65–3.65)**	1.51(0.85–2.68)
Rich	1.05(0.76–1.46)	2.40(1.53–3.76)**	0.57(0.23–1.39)	1.03(0.23–4.51)	1.15(0.74–1.80)	0.27(0.12–0.64)*	1.42(0.93–2.17)	1.81(1.02–2.23)*
Middle	0.71(0.50–1.01)	0.57(0.34–0.97)*	1.17(0.45–3.06)	0.20(0.02–2.58)	1.19(0.86–1.65)	0.46(0.29–0.73)**	3.37(2.38–4.77)*	9.07(5.78–14.25)**
Poor	0.61(0.43–0.86)*	1.08(0.67–1.76)	1.14(0.51–2.57)	0.67(0.17–2.64)	0.94(0.67–1.32)	1.50(1.05–2.16)*	0.89(0.62–1.26)	0.05(0.01–0.21)**
Poorest (Ref)								
**Education**								
No formal Education	0.87(0.58–1.31)	0.23(0.14–0.38)**	0.83(0.42–1.63)	0.31(0.08–1.19)	1.96(1.36–2.83)*	1.31(0.77–2.22)	0.53(0.35–0.79)*	0.30(0.15–0.60)**
Primary Education	1.07(0.73–1.55)	0.64(0.41–0.98)*	0.84(0.44–1.61)	1.21(0.35–4.15)	2.20(1.54–3.13)**	1.52(0.89–2.59)	0.63(0.43–0.92)*	1.17(0.70–1.95)
Secondary Education and Above (ref)								
**Occupation**								
Employed	1.73(1.20–2.48)*	1.13(0.75–1.70)	2.50(1.30–4.78)**	2.31(0.63–8.46)	0.71(0.55–0.93)*	0.61(0.45–0.84)*	2.25(1.48–3.43)**	1.07(0.61–1.90)
Unemployed(ref)								

**Dependent Variable:** Previous quit attempts in the last 12 months:—*Did not try to quit (ref);* Tried to quit without assistance; Tried to quit with assistance. Ref = Reference Category.

* = p value < 0.05,

** = p value < 0.01.

## Discussion

Significant differences in smoking cessation behavior were observed across the four countries studied. While more than 49% of the smokers reported an intention to quit smoking within 12 months following survey in Senegal, only 23.5% of the smokers from Ethiopia reported intention to quit within the said timeframe. The same was observed at 43.3% and 36.7% in Kenya and Tanzania respectively. Earlier studies on cessation behavior among smokers identified that smokers from low-income countries reported low levels of quitting compared to smokers from high income countries [25]. Among the four LMICs compared, Kenya and Senegal, the countries with greater per capita gross domestic product (GDP) had higher prevalence of quit attempts and intention to quit, compared to countries with lower percapita GDP (Ethiopia and Tanzania) [26]. Moreover, studies also suggest the impact of tobacco cessation campaigns within the country to be positively affecting its quit rates. In Senegal for example, percentage of current smokers with previous quit attempts in the last one year was found to be 53.7%, the highest among the four countries studied. The higher percentage of quit attempts can be due to the intensive smoking cessation campaign in Senegal. The 2017 WHO report on Global Tobacco Epidemic lists Senegal as the only low-income country which provides full Tobacco cessation support [22]. The 2021 WHO report ranks Senegal among the countries with complete compliance with bans on advertising, promotion & sponsorship, and adherence to smoke free laws with a full score of 10/10 [27].

The smoking cessation behavior was observed to be significantly predicted by the determinants such as gender, age, place of residence, marital status, wealth index, education level and occupational status.

While males accounted for higher percentage among the current smokers, interesting observations were made pertaining to association between gender and smoking cessation behavior. While gender was not a significant predictor for smoking cessation behavior in Senegal, female gender was significantly predicting the previous quit attempts and intention to quit in Tanzania, Kenya and Ethiopia. With respect to intention to quit it was observed that in Tanzania, females had lower odds of intention to quit within one month and next 12 months (OR = 0.11, 95% CI = 0.05–0.25 and OR = 0.60, 95% CI = 0.38–0.95 respectively) compared to that of male smokers. In Kenya however, female smokers had twice the odds of intention to quit within 12 months (OR = 2.01, 95% CI = 1.11–3.63) compared to their male counterparts. A cross sectional study comparing 607 female and 658 male smokers from Switzerland reported that a higher percentage of females perceived it difficult to quit smoking [28]. Another study from the United States reported a stronger association between knowledge of health effects of smoking and intention to quit smoking among females compared to males [29]. The findings highlight the cross-country differentials on smoking cessation behavior among male and female smokers, and suggestive gaps in awareness about tobacco indicating the need for gender specific focus on tobacco cessation measures. Concerning previous quit attempts, it was observed that compared to male smokers, female smokers had lower odds of making any previous quit attempts without taking assistance. A study based on GATS Wave 1 in India reported that males had higher odds of attempting to quit tobacco than females [30]. From the study findings it can be argued that even though males had a higher prevalence of smoking (as witnessed in Table 1) the quit behavior was also significantly high among them. Given that quit attempts are directly associated with tobacco cessation advice, it can be argued that there is a potential gap in access to tobacco cessation advice, awareness and support among females compared to males.

The age of the individual was found to be a significant predictor for smoking cessation behavior across the countries. Individuals belonging to younger age groups had higher odds of previous quit attempts and intention to quit smoking (Tanzania, Kenya and Ethiopia). These observations can be supported by a 2019 study based on international tobacco control (ITC) surveys in Kenya and Zambia [31]. The current study also found that smokers from younger age groups had a better intention to quit smoking compared to their older counterparts. A recent study based on international tobacco control four-country survey (Canada, United States, United Kingdom and Australia) reported that older smokers are more likely to be daily smokers and less likely to attempt to quit smoking [32]. The higher intention to quit among younger age groups could be explained by educational status and better awareness towards health effects of tobacco among younger age groups [33]. These observations also signify that tobacco cessation interventions targeting younger age groups in these countries can have a greater probability of success.

Interesting differences were observed with respect to the relationship between place of residence and smoking cessation behavior. Urban dwelling Kenyans & Ethiopians had greater odds of previous quit attempts in last 12 months, and intention to quit smoking in forthcoming year. Earlier studies reported that Urban Kenya has better health indicators compared to rural Kenya [34]. Another study from Brazil identified that smoking cessation was higher in urban regions and smoking prevalence decreased with urbanization [35]. In contrary to Kenya and Ethiopia, the urban dwelling individuals in Senegal and Tanzania had lower odds of having a previous quit attempt or intention to quit smoking.

Marital status was also found to be a consistent predictor for intention to quit smoking and previous quit attempts among smokers in Senegal, Kenya and Ethiopia. Married individuals had better odds of intention to quit over a 12-month horizon. Existing literature establishes a strong association between smoking cessation among smokers and smoking habits of other individuals in household [36, 37]. Studies from the United States and Japan report a positive spousal influence on abstinence from smoking and successful cessation [38, 39]. Similar observations were made in other studies in other LMIC context [15]. Occupational status was found to significantly predict previous quit attempts in Tanzania, Senegal and Ethiopia. Specifically, individuals employed in income generating activities had greater odds of attempting to quit without assistance in the last one year compared to those who were unemployed. One of the contributing aspects in this regard could be encouraging smoke free workplaces in some of the countries. For example, Senegal enacted legislations to curb indoor smoking by prohibiting tobacco smoking at workplaces [40]. A study from India reported that worksite practices were significantly correlated with quit attempts made by current smokers [41]. Another study from the US indicated that the prevalence of smoking was low in indoor workspaces which had 100% smoke free policies and cessation programmes offered by the employers [42]. These observations show that workplace specific smoke free laws work. Moreover, a recent study from India reported that having regulations on indoor smoking can significantly reduce the odds of hardening of smoking among the smokers [43]. Another interesting observation with respect to occupation status and intention to quit is case of Kenya and Tanzania. Smokers in Kenya who were employed had significantly lower odds of intending to quit smoking compared to smokers who were not employed. This finding can be explained through tobacco taxation and pricing in Kenya. Kenya has the highest percentage of taxes on cigarettes among the four countries studied. As of 2018, taxes account for 52.3% of the retail prices of most widely sold cigarettes in Kenya reducing their affordability [12]. Moreover, studies also argue that Kenya lacks strict enforcement of smoke-free work places and has poor monitoring [44], which can explain the inverse relationship between occupation status and smoking cessation behavior observed in Kenya.

Regional differences were observed with respect to Education level as a predictor. For example, Smokers without any education had significantly lower odds of intending to quit smoking within 12 months in Senegal and within one month in Ethiopia. Similarly with respect to previous quit attempts, while smokers with lower education levels had significant lower odds of attempting quitting with assistance in Tanzania and Ethiopia, they had significantly higher odds of attempting quitting in Kenya. These findings signify that smokers with higher education (secondary education and above) are keener to attempt to quit with assistance compared to those with lower education levels. A study conducted among Norwegian adults reported that higher education significantly predicts smoking cessation [45]. Another qualitative enquiry from Fiji reported that knowledge on harms of smoking was among the factors influencing smoking cessation [46]. Evidence from India indicates that a smoker’s education level is correlated with positive attitude towards smoking cessation and quit attempts [41]. The opposite results in Kenya’s context could be reflective of the regional differences in the tobacco control policies within the countries, pricing of tobacco products, availability and distribution of cessation assistance and, perceptions on effectiveness of smoking cessation [47, 48].

The influence of wealth index on smoking cessation behavior cannot be ignored. While regional differences were observed, wealth index was found to be significantly associated with smoking cessation behavior in all the four countries studied. Across the countries it was observed that smokers in better wealth index level (such as poor, middle, rich, richest) had higher odds of intention to quit and previous quit attempts compared to poorest quintile [15]. Moreover, economic status of smokers is known to interact with other vulnerabilities. A study from Serbia reported that while richest men were more likely to quit smoking, the poorest women were least likely to quit [49]. Poverty and other social vulnerabilities compromise the access to smoking cessation support. A qualitative study of smokers from marginalized communities reported that higher price of alternatives to combustible tobacco was a major hurdle for smoking cessation among nicotine dependent smokers [50]. A study from Kerala, India reported the limited availability & unaffordability of tobacco cessation medications within the health system. The study also reported that the tobacco cessation treatment regimens cost up to 13 times the median amount spent on tobacco and around 52% of non-subsistence income [51].

Smoking cessation seems to be multifactorial and influenced by socio-economic and policy dimensions. From the study findings, country specific differences in smoking cessation behavior and its predictors can be noted. The findings reflect that the impact of micro-level determinants such as education, income, wealth, occupation etc., are in turn differentiated by macro level factors such as the tobacco control policies, distribution of resources and availability of tobacco cessation opportunities [52]. The study findings reflect need for country specific approaches to effectively implement MPOWER measures.

## Conclusion

Our study findings conclude that smoking cessation behavior in the selected African countries was significantly influenced by socio-economic status, education, age and gender of the smokers. Across the four countries studied, the previous quit attempts among smokers were in the range of 39.6% to 53.7%. Around 7.6% to 15.8% of the smokers tried to quit with an assistance. No intention to quit in the next 12 months was reported by 76.5% of current smokers in Ethiopia, 56.7% in Kenya, 63.3% in Tanzania and 50.4% in Senegal. While country specific differences were observed, it could be said that female gender, poorest wealth index, unemployed and those without any formal education had least odds of undertaking previous quit attempts or having an intention to quit smoking. The results signify that socio-economic vulnerabilities aggravate the risk of NCDs attributable to smoking. Tobacco cessation interventions should focus on individuals from younger age groups and those who face the circumstances of social disadvantage. Adequate provision of smoking cessation assistance through public health system and improving awareness about them could contribute to improving quit rates. Low- and middle-income countries should also focus on making the tobacco cessation support & medications affordable which could potentially improve quit rates. Additionally, government, civil society and concerned stakeholders should push towards adherence to smokefree laws and effective mechanisms to monitor them.

## Limitations

Our study relied on secondary analysis of GATS data. Tobacco control policies in African countries are rapidly evolving to embrace the MPOWER framework. The timeliness of the GATS data included in the study might be prior to the recent developments in some countries. Additionally, the study is limited by its cross-sectional nature. Nevertheless, the study provides a representative comparison of socio-economic determinants of smoking cessation behavior across the four countries studies.

## Supporting information

S1 File (DOCX)
