# Supplementary material for: Effect of socioeconomic status on smoking cessation behavior in selected African countries: Secondary analysis of Global Adult Tobacco Survey data (2014–2018)
Source: PLoS One. 2022 Sep 19;17(9):e0274746. doi: 10.1371/journal.pone.0274746 (PMC9484673; doi:10.1371/journal.pone.0274746)
Supplement: S1 File — (DOCX) [file pone.0274746.s001.docx]

**Supplementary File 1**

**Ethiopia**

|  | **Main Effects(Model 1)** | | **Gender(Model 2)** | | **Age Group(Model 3)** | | **Residence (Model 4)** | |
| --- | --- | --- | --- | --- | --- | --- | --- | --- |
|  | **Intention to Quit** | | **Intention to Quit** | | **Intention to Quit** | | **Intention to Quit** | |
|  | Intend to Quit within a month | Intend to quit within 12 months | Intend to Quit within a month | Intend to quit within 12 months | Intend to Quit within a month | Intend to quit within 12 months | Intend to Quit within a month | Intend to quit within 12 months |
| **Gender** |  |  |  |  |  |  |  |  |
| Female | 5.56(3.00-10.30)** | 0.51(0.32-0.82)* |  |  |  |  |  |  |
| Male (ref) |  |  |  |  |  |  |  |  |
| **Age Group** |  |  |  |  |  |  |  |  |
| 15 years-35 years | 0.46(0.29-0.72)* | 16.30(6.70-39.64)* | 0.54(0.35-0.84)** | 14.93(6.17-36.15) |  |  |  |  |
| 36-50 years | 0.53(0.30-0.95)* | 7.31(2.95-18.10)* | 0.56(0.32-1.00) | 6.90(2.79-17.04) |  |  |  |  |
| 51 years and more (ref) |  |  |  |  |  |  |  |  |
| **Residence** |  |  |  |  |  |  |  |  |
| Urban | 8.26(5.09-13.39)** | 4.17(2.74-6.35) | 8.39(5.21-13.50) | 4.38(2.88-6.66) | 7.66(4.82-12.16)** | 3.69(2.51-5.43)** |  |  |
| Rural (ref) |  |  |  |  |  |  |  |  |
| **Marital Status** |  |  |  |  |  |  |  |  |
| Married | 1.80(1.08-2.98)* | 1.99(1.33-2.96)* | 2.04(1.24-3.37)** | 1.94(1.32-2.87)** | 2.62(1.62-4.25)** | 1.30(0.90-1.87) | 1.00(0.70-1.46) | 0.79(0.57-1.10) |
| Unmarried(ref) |  |  |  |  |  |  |  |  |
| **Wealth Index** |  |  |  |  |  |  |  |  |
| Richest | 10.59(6.11-18.33)* | 1.24(0.71-2.18) | 9.18(5.35-15.76)** | 1.55(0.90-2.68) | 10.50(6.11-18.05)** | 1.10(0.65-1.85) | 12.18(7.61-19.47) | 1.25(0.77-2.04) |
| Rich | 14.83(7.78-28.28)* | 3.12(1.94-4.99) | 11.72(6.23-22.04)** | 3.89(2.48-6.10)** | 10.83(5.95-19.69)** | 3.09(2.02-4.73)** | 5.97(3.59-9.94) | 2.35(1.56-3.48)** |
| Middle | 3.12(1.79-5.42)* | 0.94(0.60-1.48) | 3.23(1.88-5.56)** | 1.08(0.70-1.69) | 2.99(1.74-5.12)** | 0.93(0.61-1.43) | 2.34(1.50-3.65) | 0.52(0.34-0.77)** |
| Poor | 2.50(1.34-4.68)* | 0.81(0.51-1.29) | 2.26(1.22-4.17)** | 0.92(0.58-1.45) | 2.04(1.12-3.74)* | 0.65(0.42-1.00) | 5.45(3.54-8.39) | 0.90(0.62-1.29) |
| Poorest (ref) |  |  |  |  |  |  |  |  |
| **Education** |  |  |  |  |  |  |  |  |
| No formal Education | 0.19(0.07-0.52)** | 2.15(1.26-3.66)* | 0.24(0.09-0.63) | 1.95(1.16-3.29) | 0.25(0.09-0.66)** | 1.31(0.81-2.12) | 0.54(0.34-0.85)** | 0.54(0.38-0.76) |
| Primary Education | 7.08(4.21-11.92)* | 0.94(0.58-1.56) | 6.72(3.90-11.361) | 0.99(0.61-1.61) | 6.56(3.93-10.91)** | 0.71(0.57-1.46) | 3.49(2.47-4.93)** | 0.52(0.36-0.73) |
| Secondary Education and Above (ref) |  |  |  |  |  |  |  |  |
| **Occupation** |  |  |  |  |  |  |  |  |
| Employed | 2.78(1.44-5.36)** | 0.52(0.34-0.78)** | 1.38(0.78-2.44) | 0.64(0.44-0.93)* | 1.26(0.73-2.18) | 3.60(2.44-5.31)** | 1.32(0.84-2.06) | 0.90(0.62-1.29) |
| Unemployed(ref) |  |  |  |  |  |  |  |  |

**Dependent Variable:** Intention to quit smoking in the next 12 months: - *No intention to quit (ref);* Intend to quit within one month; Intention to quit in next 12 months. Ref = Reference Category. *= p value < 0.05, **= p value < 0.01.

|  | **Main Effects(Model 1)** | | **Gender(Model 2)** | | **Age Group(Model 3)** | | **Residence (Model 4)** | |
| --- | --- | --- | --- | --- | --- | --- | --- | --- |
|  | **Previous Quit Attempts** | | **Previous Quit Attempts** | | **Previous Quit Attempts** | | **Previous Quit Attempts** | |
|  | Tried to quit without assistance | Tried to quit with assistance | Tried to quit without assistance | Tried to quit with assistance | Tried to quit without assistance | Tried to quit with assistance | Tried to quit without assistance | Tried to quit with assistance |
| **Gender** |  |  |  |  |  |  |  |  |
| Female | 0.59(0.40-0.87)* | 0.014(0.002-0.085)** |  |  |  |  |  |  |
| Male (ref) |  |  |  |  |  |  |  |  |
| **Age Group** |  |  |  |  |  |  |  |  |
| 15 years-35 years | 1.34(0.97-1.86) | 20.06(8.24-48.84)* | 1.25(0.90-1.74) | 15.29(6.32-37.00)** |  |  |  |  |
| 36-50 years | 1.50(1.03-2.18)* | 6.41(2.41-17  .06)** | 1.47(1.01-2.13)* | 5.86(2.21-15.54)** |  |  |  |  |
| 51 years and more (ref) |  |  |  |  |  |  |  |  |
| **Residence** |  |  |  |  |  |  |  |  |
| Urban | 2.72(1.97-3.75)* | 3.55(2.27-5.56)* | 2.76(2.00-3.82)** | 3.58(2.32-5.54)** | 2.81(2.04-3.87)** | 3.39(2.24-5.12)** |  |  |
| Rural (ref) |  |  |  |  |  |  |  |  |
| **Marital Status** |  |  |  |  |  |  |  |  |
| Married | 1.65(1.16-2.32)* | 2.12(1.31-3.42)* | 1.63(1.16-2.30)** | 1.95(1.24-3.07)** | 1.57(1.13-2.17)** | 1.24(0.81-1.92) | 1.99(1.49-2.65)** | 0.71(0.49-1.04) |
| Unmarried(ref) |  |  |  |  |  |  |  |  |
| **Wealth Index** |  |  |  |  |  |  |  |  |
| Richest | 2.45(1.65-3.65)** | 1.51(0.85-2.68) | 2.73(1.84-4.04)** | 1.98(1.12-3.49)* | 2.66(1.81-3.90)** | 1.41(0.82-2.40) | 3.11(2.19-4.41)** | 1.62(0.96-2.72) |
| Rich | 1.42(0.93-2.17) | 1.81(1.02-2.23)* | 1.63(1.08-2.47)* | 2.39(1.35-4.23)** | 1.76(1.17-2.64)** | 1.87(1.08-3.23)** | 1.30(0.90-1.89) | 1.65(1.00-2.74) |
| Middle | 3.37(2.38-4.77)* | 9.07(5.78-14.25)** | 3.44(2.44-4.86)** | 8.50(5.49-13.16)** | 3.50(2.49-4.93)** | 7.92(5.24-11.97)** | 3.41(2.60-4.47)** | 4.92(3.38-7.16)** |
| Poor | 0.89(0.62-1.26) | 0.05(0.01-0.21)** | 0.96(0.68-1.36) | 0.05(0.01-0.26)** | 0.98(0.69-1.38) | 0.06(0.01-0.26)** | 1.66(1.27-2.18)** | 0.26(0.10-0.50)** |
| Poorest (Ref) |  |  |  |  |  |  |  |  |
| **Education** |  |  |  |  |  |  |  |  |
| No formal Education | 0.53(0.35-0.79)* | 0.30(0.15-0.60)** | 0.49(0.33-0.75)** | 0.24(0.12-0.47)** | 0.51(0.34-0.76)** | 0.19(0.10-0.35) | 0.19(0.14-0.25)** | 0.12(0.07-0.21)** |
| Primary Education | 0.63(0.43-0.92)* | 1.17(0.70-1.95) | 0.65(0.44-0.95)* | 1.14(0.70-1.85) | 0.63(0.43-0.91)* | 1.10(0.69-1.77) | 0.25(0.20-0.35)** | 0.96(0.66-1.39) |
| Secondary Education and Above (ref) |  |  |  |  |  |  |  |  |
| **Occupation** |  |  |  |  |  |  |  |  |
| Employed | 2.25(1.48-3.43)** | 1.07(0.61-1.90) | 2.75(1.85-4.10)** | 2.07(1.24-3.47)** | 2.79(1.87-4.15)** | 2.06(1.24-3.43)** | 3.51(2.49-4.95)** | 1.98(1.24-3.17)** |
| Unemployed(ref) |  |  |  |  |  |  |  |  |

**Dependent Variable:** Previous quit attempts in the last 12 months: - *Did not try to quit (ref);* Tried to quit without assistance; Tried to quit with assistance . Ref = Reference Category. *= p value < 0.05, **= p value < 0.01.

**Kenya**

|  | **Main Effects(Model 1)** | | **Gender(Model 2)** | | **Age Group(Model 3)** | | **Residence (Model 4)** | |
| --- | --- | --- | --- | --- | --- | --- | --- | --- |
|  | **Intention to Quit** | | **Intention to Quit** | | **Intention to Quit** | | **Intention to Quit** | |
|  | Intend to Quit within a month | Intend to quit within 12 months | Intend to Quit within a month | Intend to quit within 12 months | Intend to Quit within a month | Intend to quit within 12 months | Intend to Quit within a month | Intend to quit within 12 months |
| **Gender** |  |  |  |  |  |  |  |  |
| Female | 1.14(0.63-2.01) | 2.01(1.11-3.63)* |  |  |  |  |  |  |
| Male (ref) |  |  |  |  |  |  |  |  |
| **Age Group** |  |  |  |  |  |  |  |  |
| 15 years-35 years | 1.50(1.04-2.14)* | 1.09(0.72-1.67) | 1.49(1.05-2.13)* | 0.99(0.65-1.49) |  |  |  |  |
| 36-50 years | 1.55(1.10-2.19)* | 1.70(1.15-2.53)* | 1.55(1.10-2.16)* | 1.52(1.04-2.21)* |  |  |  |  |
| 51 years and more (ref) |  |  |  |  |  |  |  |  |
| **Residence** |  |  |  |  |  |  |  |  |
| Urban | 1.51 (1.14-2.00)* | 1.24 (0.89-2.69) | 1.52(1.14-2.02)** | 3.93(2.87-5.39)** | 1.51(1.14-2.00)** | 3.79(2.77-5.18)** |  |  |
| Rural (ref) |  |  |  |  |  |  |  |  |
| **Marital Status** |  |  |  |  |  |  |  |  |
| Married | 1.76(1.30-2.41)* | 1.05(0.74-1.51) | 1.76(1.29-2.41)** | 1.08(0.75-1.54) | 1.64(1.25-2.16)** | 1.23(0.90-1.67) | 1.58(1.21-2.08)** | 1.12(0.82-1.52) |
| Unmarried(ref) |  |  |  |  |  |  |  |  |
| **Wealth Index** |  |  |  |  |  |  |  |  |
| Richest | 2.46(1.52-3.97)* | 2.40(1.37-4.20)** | 2.46(1.53-3.96)** | 2.69(1.55-4.67)** | 2.61(1.63-4.19)** | 2.76(1.59-4.78)** | 2.75(1.71-4.41)** | 3.34(1.96-5.71)** |
| Rich | 0.83(0.49-1.39) | 1.48(0.80-2.73) | 0.83(0.49-1.39) | 1.52(0.83-2.80) | 0.81(0.49-1.35) | 1.58(0.86-2.89) | 0.80(0.48-1.33) | 1.56(0.86-2.82) |
| Middle | 0.89(0.63-1.28) | 1.94(1.25-3.00)* | 0.85(0.60-1.21) | 1.98(1.28-3.06)** | 0.90(0.64-1.28) | 2.02(1.30-3.12)** | 0.85(0.60-1.21) | 1.67(1.09-2.56)* |
| Poor | 2.09(1.50-2.93)* | 4.03(2.67-6.09)* | 2.10(1.51-2.93)** | 4.20(2.78-6.34)** | 2.10(1.49-2.89)** | 4.22(2.80-6.36)** | 1.95(1.40-2.70)** | 3.40(2.29-5.07)** |
| Poorest (ref) |  |  |  |  |  |  |  |  |
| **Education** |  |  |  |  |  |  |  |  |
| No formal Education | 2.60(1.67-4.05)** | 1.77(1.13-2.77)* | 2.61(1.68-4.05)** | 1.94(1.25-3.02) | 2.67(1.73-4.12)** | 1.96(1.26-3.05)** | 2.34(1.53-3.57)** | 1.31(0.87-1.99) |
| Primary Education | 3.55(2.32-5.43)** | 1.14(0.72-1.78) | 3.57(2.34-5.44)** | 1.20(0.77-1.88) | 3.98(2.63-6.02)** | 1.30(0.84-2.01) | 3.68(2.45-5.55)** | 1.04(0.68-1.58) |
| Secondary Education and Above (ref) |  |  |  |  |  |  |  |  |
| **Occupation** |  |  |  |  |  |  |  |  |
| Employed | 0.60(0.45-0.79)* | 0.53(0.38-0.74)* | 0.63(0.48-0.82)** | 0.64(0.47-0.87)** | 1.57(1.20-2.05)** | 1.98(1.45-2.72)** | 1.47(1.13-1.91)** | 1.54(1.14-2.07)** |
| Unemployed(ref) |  |  |  |  |  |  |  |  |

**Dependent Variable:** Intention to quit smoking in the next 12 months: - *No intention to quit (ref);* Intend to quit within one month; Intention to quit in next 12 months. Ref = Reference Category. *= p value < 0.05, **= p value < 0.01.

|  | **Main Effects(Model 1)** | | **Gender(Model 2)** | | **Age Group(Model 3)** | | **Residence (Model 4)** | |
| --- | --- | --- | --- | --- | --- | --- | --- | --- |
|  | **Previous Quit Attempts** | | **Previous Quit Attempts** | | **Previous Quit Attempts** | | **Previous Quit Attempts** | |
|  | Tried to quit without assistance | Tried to quit with assistance | Tried to quit without assistance | Tried to quit with assistance | Tried to quit without assistance | Tried to quit with assistance | Tried to quit without assistance | Tried to quit with assistance |
| **Gender** |  |  |  |  |  |  |  |  |
| Female | 0.49(0.27-0.90)* | 1.47(0.83-2.61) |  |  |  |  |  |  |
| Male (ref) |  |  |  |  |  |  |  |  |
| **Age Group** |  |  |  |  |  |  |  |  |
| 15 years-35 years | 1.18(0.85-1.63) | 0.90(0.59-1.38) | 1.23(0.89-1.71) | 0.84(0.56-1.26) |  |  |  |  |
| 36-50 years | 1.36(1.01-1.84)* | 1.02(0.67-1.55) | 1.46(1.08-1.97)* | 0.94(0.63-1.40) |  |  |  |  |
| 51 years and more (ref) |  |  |  |  |  |  |  |  |
| **Residence** |  |  |  |  |  |  |  |  |
| Urban | 1.65(1.28-2.13)* | 1.28(0.90-1.80) | 1.60(1.25-2.06)** | 1.31(0.93-1.84) | 1.58(1.23-2.02)** | 1.31(0.93-1.84) |  |  |
| Rural (ref) |  |  |  |  |  |  |  |  |
| **Marital Status** |  |  |  |  |  |  |  |  |
| Married | 1.62(1.21-2.17)* | 0.50(0.35-0.71)** | 1.59(1.19-2.13)** | 0.50(0.56-1.26)** | 1.60(1.23-2.08)** | 0.54(0.40-0.73)** | 1.55(1.20-2.01)** | 0.53(0.39-0.72)** |
| Unmarried(ref) |  |  |  |  |  |  |  |  |
| **Wealth Index** |  |  |  |  |  |  |  |  |
| Richest | 2.78(1.79-4.23)** | 0.61(0.32-1.15) | 2.56(1.67-3.93)** | 0.66(0.35-1.25)** | 2.67(1.74-4.09)** | 0.64(0.34-1.21) | 2.87(1.87-4.39)** | 0.67(0.36-1.26) |
| Rich | 1.15(0.74-1.80) | 0.27(0.12-0.64)* | 1.13(0.73-1.77)** | 0.28(0.12-0.65)** | 1.13(0.73-1.75) | 0.28(0.12-0.66)** | 1.13(0.73-1.75) | 0.28(0.12-0.66)** |
| Middle | 1.19(0.86-1.65) | 0.46(0.29-0.73)** | 1.17(0.85-1.61)** | 0.47(0.30-0.74)** | 1.17(0.85-1.61) | 0.46(0.29-0.74)** | 1.11(0.81-1.52) | 0.45(0.29-0.71)** |
| Poor | 0.94(0.67-1.32) | 1.50(1.05-2.16)* | 0.91(0.65-1.27)** | 1.54(1.07-2.21)** | 0.91(0.65-1.27) | 1.53(1.07-2.20)** | 0.87(0.62-1.21) | 1.49(1.04-2.13)* |
| Poorest (Ref) |  |  |  |  |  |  |  |  |
| **Education** |  |  |  |  |  |  |  |  |
| No formal Education | 1.96(1.36-2.83)* | 1.31(0.77-2.22) | 1.86(1.30-2.67)** | 1.41(0.83-2.39) | 1.88(1.31-2.69)** | 1.40(0.82-2.38) | 1.63(1.15-2.31)** | 1.29(0.77-2.17) |
| Primary Education | 2.20(1.54-3.13)** | 1.52(0.89-2.59) | 2.12(1.49-3.01)** | 1.59(0.93-2.71) | 2.32(1.65-3.27)** | 1.57(0.93-2.66) | 2.12(1.52-2.97)** | 1.50(0.89-2.53) |
| Secondary Education and Above (ref) |  |  |  |  |  |  |  |  |
| **Occupation** |  |  |  |  |  |  |  |  |
| Employed | 0.71(0.55-0.93)* | 0.61(0.45-0.84)* | 1.32(1.02-1.72)* | 1.68(1.23-2.30)** | 1.27(0.98-1.84) | 1.74(1.28-2.36)** | 1.18(0.92-1.52) | 1.67(1.23-2.26)** |
| Unemployed(ref) |  |  |  |  |  |  |  |  |

**Dependent Variable:** Previous quit attempts in the last 12 months: - *Did not try to quit (ref);* Tried to quit without assistance; Tried to quit with assistance . Ref = Reference Category. *= p value < 0.05, **= p value < 0.01.

**Senegal**

|  | **Main Effects(Model 1)** | | **Gender(Model 2)** | | **Age Group(Model 3)** | | **Residence (Model 4)** | |
| --- | --- | --- | --- | --- | --- | --- | --- | --- |
|  | **Intention to Quit** | | **Intention to Quit** | | **Intention to Quit** | | **Intention to Quit** | |
|  | Intend to Quit within a month | Intend to quit within 12 months | Intend to Quit within a month | Intend to quit within 12 months | Intend to Quit within a month | Intend to quit within 12 months | Intend to Quit within a month | Intend to quit within 12 months |
| **Gender** |  |  |  |  |  |  |  |  |
| Female | 0.86(0.25-2.93) | 0.23(0.02-2.69) |  |  |  |  |  |  |
| Male (ref) |  |  |  |  |  |  |  |  |
| **Age Group** |  |  |  |  |  |  |  |  |
| 15 years-35 years | 1.64(0.82-3.29) | 0.83(0.38-1.81) | 1.64(0.82-3.29) | 0.82(0.38-1.77) |  |  |  |  |
| 36-50 years | 0.81(0.39-1.67) | 0.85(0.40-1.81) | 0.80(0.39-1.67) | 0.86(0.41-1.82) |  |  |  |  |
| 51 years and more (ref) |  |  |  |  |  |  |  |  |
| **Residence** |  |  |  |  |  |  |  |  |
| Urban | 0.60(0.31-1.17) | 0.88(0.41-1.91) | 0.60(0.31-1.18) | 0.88(0.41-1.90) | 0.58(0.30-1.13) | 0.87(0.40-1.88) |  |  |
| Rural (ref) |  |  |  |  |  |  |  |  |
| **Marital Status** |  |  |  |  |  |  |  |  |
| Married | 0.78(0.42-1.42) | 1.31(0.66-2.57) | 0.77(0.42-1.42) | 1.28(0.65-2.50) | 0.63(0.36-1.11) | 1.34(0.71-2.53) | 0.40(0.24-0.67)** | 1.06(0.59-1.90) |
| Unmarried(ref) |  |  |  |  |  |  |  |  |
| **Wealth Index** |  |  |  |  |  |  |  |  |
| Richest | 1.28(0.40-4.07) | 1.22(0.38-3.90) | 1.28(0.40-4.06) | 1.25(0.40-3.93) | 1.27(0.41-3.97) | 1.26(0.40-4.02) | 0.69(0.26-1.80) | 1.16(0.43-3.07) |
| Rich | 1.89(0.67-5.29) | 1.28(0.43-3.79) | 1.89(0.67-5.29) | 1.33(0.45-3.93) | 1.89(0.68-5.26) | 1.33(0.45-3.94) | 0.93(0.39-2.21) | 1.06(0.42-2.68) |
| Middle | 4.38(1.43-13.43)* | 1.72(0.49-5.94) | 4.32(1.41-13.19)** | 1.67(0.49-5.70) | 3.95(1.31-11.89)* | 1.65(0.48-5.60) | 3.79(1.45-9.91)** | 2.29(0.78-6.71) |
| Poor | 3.55(1.39-9.08)* | 0.92(0.32-2.69) | 3.52(1.38-8.96)** | 0.87(0.30-2.53) | 3.45(1.37-8.70)** | 0.85(0.29-2.47) | 2.52(1.11-5.71)** | 1.02(0.39-2.66) |
| Poorest (ref) |  |  |  |  |  |  |  |  |
| **Education** |  |  |  |  |  |  |  |  |
| No formal Education | 0.85(0.38-1.92) | 0.45(0.20-0.99)* | 0.85(0.38-1.91) | 0.46(0.21-1.01) | 0.88(0.40-1.95) | 0.47(0.22-1.02) | 0.80(0.38-1.66) | 0.40(0.19-0.82)* |
| Primary Education | 1.22(0.56-2.65) | 0.58(0.27-1.24) | 1.21(0.56-2.63) | 0.59(0.28-1.25) | 1.16(0.54-2.45) | 0.59(0.28-1.25) | 1.07(0.51-2.24) | 0.82(0.40-1.64) |
| Secondary Education and Above (ref) |  |  |  |  |  |  |  |  |
| **Occupation** |  |  |  |  |  |  |  |  |
| Employed | 1.43(0.70-2.96) | 1.07(0.74-3.78) | 1.45(0.72-2.94) | 1.78(0.80-3.94) | 0.87(0.40-1.88) | 1.74(0.80-3.75) | 1.58(0.84-2.98) | 1.55(0.79-3.06) |
| Unemployed(ref) |  |  |  |  |  |  |  |  |

**Dependent Variable:** Intention to quit smoking in the next 12 months: - *No intention to quit (ref);* Intend to quit within one month; Intention to quit in next 12 months. Ref = Reference Category. *= p value < 0.05, **= p value < 0.01.

|  | **Main Effects(Model 1)** | | **Gender(Model 2)** | | **Age Group(Model 3)** | | **Residence (Model 4)** | |
| --- | --- | --- | --- | --- | --- | --- | --- | --- |
|  | **Previous Quit Attempts** | | **Previous Quit Attempts** | | **Previous Quit Attempts** | | **Previous Quit Attempts** | |
|  | Tried to quit without assistance | Tried to quit with assistance | Tried to quit without assistance | Tried to quit with assistance | Tried to quit without assistance | Tried to quit with assistance | Tried to quit without assistance | Tried to quit with assistance |
| **Gender** |  |  |  |  |  |  |  |  |
| Female | 0.94(0.30-3.00) | 0.96(0.29-2.59) |  |  |  |  |  |  |
| Male (ref) |  |  |  |  |  |  |  |  |
| **Age Group** |  |  |  |  |  |  |  |  |
| 15 years-35 years | 0.97(0.52-1.79) | 4.43(1.17-16.73) | 0.97(0.52-1.79) | 4.31(1.14-16.30)* |  |  |  |  |
| 36-50 years | 0.93(0.50-1.73) | 1.47(0.36-3.98) | 0.93(0.50-1.72) | 1.50(0.37-6.08) |  |  |  |  |
| 51 years and more (ref) |  |  |  |  |  |  |  |  |
| **Residence** |  |  |  |  |  |  |  |  |
| Urban | 0.73(0.40-1.35) | 0.28(0.09-0.91)* | 0.74(0.40-1.35) | 0.26(0.08-0.83)* | 0.74(0.40-1.35) | 0.27(0.09-0.82)* |  |  |
| Rural (ref) |  |  |  |  |  |  |  |  |
| **Marital Status** |  |  |  |  |  |  |  |  |
| Married | 0.72(0.42-1.23) | 3.48(1.06-11.43)* | 0.72(0.42-1.22) | 3.26(1.00-10.61) | 0.71(0.43-1.18) | 1.94(0.66-5.71) | 0.75(0.48-1.18) | 1.09(0.45-2.64) |
| Unmarried(ref) |  |  |  |  |  |  |  |  |
| **Wealth Index** |  |  |  |  |  |  |  |  |
| Richest | 0.73(0.28-1.90) | 0.50(0.08-3.03) | 0.73(0.28-1.89) | 0.51(0.09-2.97) | 0.73(0.28-1.89) | 0.51(0.09-2.83) | 0.81(0.37-1.77) | 0.23(0.05-1.06) |
| Rich | 0.57(0.23-1.39) | 1.03(0.23-4.51) | 0.57(0.23-1.39) | 1.11(0.26-4.72) | 0.57(0.23-1.38) | 1.12(0.27-4.62) | 0.58(0.28-1.20) | 0.51(0.15-1.77) |
| Middle | 1.17(0.45-3.06) | 0.20(0.02-2.58) | 1.16(0.44-3.03) | 0.20(0.16-2.56) | 1.14(0.44-2.97) | 0.20(0.02-2.46) | 1.67(0.73-3.83) | 0.14(0.01-1.65) |
| Poor | 1.14(0.51-2.57) | 0.67(0.17-2.64) | 1.13(0.50-2.54) | 0.60(0.15-2.32) | 1.12(0.50-2.51) | 0.70(0.19-2.67) | 1.24(0.61-2.53) | 0.98(0.30-3.14) |
| Poorest (Ref) |  |  |  |  |  |  |  |  |
| **Education** |  |  |  |  |  |  |  |  |
| No formal Education | 0.83(0.42-1.63) | 0.31(0.08-1.19) | 0.83(0.42-1.63) | 0.34(0.09-1.27) | 0.84(0.43-1.64) | 0.34(0.10-1.19) | 1.06(0.57-1.95) | 0.49(0.16-1.51) |
| Primary Education | 0.84(0.44-1.61) | 1.21(0.35-4.15) | 0.84(0.43-1.61) | 1.24(0.37-4.12) | 0.84(0.44-1.61) | 1.06(0.33-3.43) | 1.11(0.60-2.05) | 0.93(0.30-2.87) |
| Secondary Education and Above (ref) |  |  |  |  |  |  |  |  |
| **Occupation** |  |  |  |  |  |  |  |  |
| Employed | 2.50(1.30-4.78)** | 2.31(0.63-8.46) | 2.51(1.33-4.76)** | 2.52(0.71-8.93) | 2.47(1.33-4.61)** | 2.24(0.66-7.63) | 1.83(1.07-3.13)* | 2.68(0.80-8.94) |
| Unemployed(ref) |  |  |  |  |  |  |  |  |

**Dependent Variable:** Previous quit attempts in the last 12 months: - *Did not try to quit (ref);* Tried to quit without assistance; Tried to quit with assistance . Ref = Reference Category. *= p value < 0.05, **= p value < 0.01.

**Tanzania**

|  | **Main Effects(Model 1)** | | **Gender(Model 2)** | | **Age Group(Model 3)** | | **Residence (Model 4)** | |
| --- | --- | --- | --- | --- | --- | --- | --- | --- |
|  | **Intention to Quit** | | **Intention to Quit** | | **Intention to Quit** | | **Intention to Quit** | |
|  | Intend to Quit within a month | Intend to quit within 12 months | Intend to Quit within a month | Intend to quit within 12 months | Intend to Quit within a month | Intend to quit within 12 months | Intend to Quit within a month | Intend to quit within 12 months |
| **Gender** |  |  |  |  |  |  |  |  |
| Female | 0.11(0.05-0.25)** | 0.60(0.38-0.95)* |  |  |  |  |  |  |
| Male (ref) |  |  |  |  |  |  |  |  |
| **Age Group** |  |  |  |  |  |  |  |  |
| 15 years-35 years | 0.90(0.64-1.28) | 1.78(1.28-2.42)* | 1.11(0.79-1.57) | 1.92(1.40-2.63)** |  |  |  |  |
| 36-50 years | 1.17(0.85-1.61) | 1.22(0.89-1.68) | 1.35(0.99-1.85) | 1.28(0.94-1.75) |  |  |  |  |
| 51 years and more (ref) |  |  |  |  |  |  |  |  |
| **Residence** |  |  |  |  |  |  |  |  |
| Urban | 0.26(0.18-0.38)* | 0.70(0.53-0.93)* | 0.27(0.19-0.39)** | 0.71(0.54-0.94)* | 0.28(0.19-0.40)** | 0.69(0.52-0.91)** |  |  |
| Rural (ref) |  |  |  |  |  |  |  |  |
| **Marital Status** |  |  |  |  |  |  |  |  |
| Married | 1.51(1.00-2.27) | 1.34(0.89-2.02) | 1.63(1.09-2.44)* | 1.50(1.00-2.26) | 1.69(1.14-2.50)* | 1.31(0.88-1.96) | 2.02(1.37-2.98)** | 1.43(0.96-2.12) |
| Unmarried(ref) |  |  |  |  |  |  |  |  |
| **Wealth Index** |  |  |  |  |  |  |  |  |
| Richest | 1.32(0.81-2.16) | 1.56(1.04-2.34)* | 1.20(0.79-1.82) | 1.58(1.05-2.37)* | 1.23(0.81-1.86) | 1.57(1.05-2.36)* | 0.80(0.54-1.12) | 1.33(0.90-1.96) |
| Rich | 0.79(0.54-1.19) | 1.08(0.71-1.65) | 0.73(0.48-1.12) | 1.11(0.73-1.68) | 0.75(0.49-1.15) | 1.08(0.71-1.64) | 0.67(0.44-1.02) | 0.99(0.66-1.49) |
| Middle | 1.15(0.76-1.72) | 1.67(1.12-2.51)* | 1.23(0.82-1.85) | 1.73(1.16-2.59)** | 1.30(0.87-1.93) | 1.61(1.09-2.40)* | 0.98(0.67-1.44) | 1.37(0.93-2.00) |
| Poor | 0.80(0.53-1.20) | 1.23(0.83-1.82) | 0.85(0.57-1.27) | 1.31(0.88-1.94) | 0.87(0.58-1.30) | 1.32(0.90-1.96) | 0.84(0.57-1.25) | 1.27(0.87-1.87) |
| Poorest (ref) |  |  |  |  |  |  |  |  |
| **Education** |  |  |  |  |  |  |  |  |
| No formal Education | 1.60(0.92-2.80) | 1.69(1.10-2.60)* | 1.33(0.77-2.28) | 1.63(1.06-2.51)* | 1.34(0.79-2.28) | 1.38(0.91-2.11) | 1.92(1.16-3.16)* | 1.56(1.04-2.34)* |
| Primary Education | 1.62(0.95-2.77) | 1.50(1.00-2.26) | 1.28(0.94-1.75) | 1.52(1.01-2.30)* | 1.58(0.95-2.64) | 1.27(0.85-1.88) | 2.04(1.26-3.30)** | 1.37(0.93-2.02) |
| Secondary Education and Above (ref) |  |  |  |  |  |  |  |  |
| **Occupation** |  |  |  |  |  |  |  |  |
| Employed | 0.62(0.43-0.90)* | 1.04(0.71-1.53) | 0.72(0.50-1.02) | 1.05(0.72-1.53) | 0.72(0.51-1.03) | 1.10(0.76-1.60) | 0.73(0.52-1.02) | 1.13(0.79-1.62) |
| Unemployed(ref) |  |  |  |  |  |  |  |  |

**Dependent Variable:** Intention to quit smoking in the next 12 months: - *No intention to quit (ref);* Intend to quit within one month; Intention to quit in next 12 months. Ref = Reference Category. *= p value < 0.05, **= p value < 0.01.

|  | **Main Effects(Model 1)** | | **Gender(Model 2)** | | **Age Group(Model 3)** | | **Residence (Model 4)** | |
| --- | --- | --- | --- | --- | --- | --- | --- | --- |
|  | **Previous Quit Attempts** | | **Previous Quit Attempts** | | **Previous Quit Attempts** | | **Previous Quit Attempts** | |
|  | Tried to quit without assistance | Tried to quit with assistance | Tried to quit without assistance | Tried to quit with assistance | Tried to quit without assistance | Tried to quit with assistance | Tried to quit without assistance | Tried to quit with assistance |
| **Gender** |  |  |  |  |  |  |  |  |
| Female | 0.48(0.31-0.75)** | 0.72(0.41-1.24) |  |  |  |  |  |  |
| Male (ref) |  |  |  |  |  |  |  |  |
| **Age Group** |  |  |  |  |  |  |  |  |
| 15 years-35 years | 1.65(1.24-2.20)* | 1.10(0.75-1.62) | 1.84(1.38-2.44)** | 1.24(0.85-1.82) |  |  |  |  |
| 36-50 years | 1.65(1.25-2.19)* | 1.90(1.34-2.70) | 1.75(1.32-2.32)** | 2.01(1.42-2.85)** |  |  |  |  |
| 51 years and more (ref) |  |  |  |  |  |  |  |  |
| **Residence** |  |  |  |  |  |  |  |  |
| Urban | 0.81(0.63-1.05) | 0.61(0.44-0.84)** | 0.88(0.68-1.14) | 0.64(0.46-0.89)** | 0.89(0.69-1.15) | 0.66(0.48-0.92)** |  |  |
| Rural (ref) |  |  |  |  |  |  |  |  |
| **Marital Status** |  |  |  |  |  |  |  |  |
| Married | 0.80(0.54-1.20) | 1.03(0.66-1.61) | 0.80(0.54-1.19) | 1.06(0.67-1.66) | 0.76(0.51-1.12) | 1.12(0.72-1.75) | 0.79(0.54-1.17) | 1.23(0.79-1.91) |
| Unmarried(ref) |  |  |  |  |  |  |  |  |
| **Wealth Index** |  |  |  |  |  |  |  |  |
| Richest | 0.89(0.60-1.32) | 0.80(0.46-1.39) | 0.75(0.53-1.06) | 0.97(0.59-1.58) | 0.78(0.55-1.10) | 1.05(0.65-1.71) | 0.74(0.53-1.04) | 0.84(0.53-1.34) |
| Rich | 1.05(0.76-1.46) | 2.40(1.53-3.76)** | 1.46(1.02-2.10)** | 3.31(2.06-5.31)** | 1.48(1.03-2.11)* | 3.54(2.22-1.15)** | 1.35(0.95-1.92) | 2.93(1.86-4.62)** |
| Middle | 0.71(0.50-1.01) | 0.57(0.34-0.97)* | 0.73(0.51-1.03) | 0.58(0.35-0.99)* | 0.74(0.53-1.04) | 0.69(0.41-1.15) | 0.69(0.50-0.97)* | 0.57(0.35-0.95)* |
| Poor | 0.61(0.43-0.86)* | 1.08(0.67-1.76) | 0.63(0.45-0.89)** | 1.11(0.69-1.80) | 0.66(0.47-0.94)* | 1.22(0.76-1.97) | 0.65(0.46-0.92)* | 1.12(0.71-1.78) |
| Poorest (Ref) |  |  |  |  |  |  |  |  |
| **Education** |  |  |  |  |  |  |  |  |
| No formal Education | 0.87(0.58-1.31) | 0.23(0.14-0.38)** | 0.80(0.54-1.18) | 0.26(0.16-0.42)** | 0.73(0.50-1.07) | 0.27(0.17-0.43)** | 0.75(0.52-1.08) | 0.30(0.20-0.47)** |
| Primary Education | 1.07(0.73-1.55) | 0.64(0.41-0.98)* | 1.08(0.74-1.55) | 0.78(0.49-1.15) | 1.00(0.70-1.42) | 0.85(0.56-1.27) | 1.00(0.71-1.42) | 0.90(0.61-1.33) |
| Secondary Education and Above (ref) |  |  |  |  |  |  |  |  |
| **Occupation** |  |  |  |  |  |  |  |  |
| Employed | 1.73(1.20-2.48)* | 1.13(0.75-1.70) | 1.84(1.28-2.64)** | 1.16(0.77-1.73) | 1.95(1.37-2.79)** | 1.19(0.80-1.77) | 1.95(1.38-2.76)** | 1.11(0.75-1.64) |
| Unemployed(ref) |  |  |  |  |  |  |  |  |

**Dependent Variable:** Previous quit attempts in the last 12 months: - *Did not try to quit (ref);* Tried to quit without assistance; Tried to quit with assistance . Ref = Reference Category. *= p value < 0.05, **= p value < 0.01.
